# Clinical characteristics of patients with ureteral endometriosis and severe renal impairment identified by diuretic renal dynamic imaging

**DOI:** 10.3389/fmed.2026.1905000

**Published:** 2026-07-27

**Authors:** Deyu Zhang, Yan Huang, Yingfang Zhou, Chao Peng

**Affiliations:** First Hospital, Peking University, Beijing, China

**Keywords:** diuretic renal dynamic imaging, hydronephrosis, nephrectomy, severe renal impairment, ureteral endometriosis

## Abstract

**Purpose:**

This study aimed to summarize the clinical characteristics, surgical management, and follow-up outcomes of patients with severe renal impairment caused by ureteral endometriosis.

**Methods:**

We conducted a retrospective analysis of the clinical data of 15 patients with severe renal impairment caused by ureteral endometriosis treated at the First Hospital of Peking University between January 2000 and January 2022.

**Results:**

Among the 15 patients, 3 (20.0%) were asymptomatic, whereas 12 (80.0%) reported endometriosis-related pain and/or urinary symptoms. Seven patients (46.7%) reported lumbar discomfort. All patients underwent preoperative diuretic renography, which showed an affected-kidney glomerular filtration rate (GFR) of <15 mL/min in all cases. Despite severe impairment of the affected kidney, only one patient had elevated serum creatinine levels, suggesting that serum creatinine may fail to detect severe unilateral renal impairment when contralateral renal function is preserved. Eight patients (53.3%) underwent ipsilateral nephrectomy, four (26.7%) underwent ureteroneocystostomy, and three (20.0%) underwent ureterolysis. During a median follow-up of 61 months, the majority of patients’ symptoms improved. Among the seven patients who underwent kidney function-preserving procedures, hydronephrosis disappeared in six patients and significantly improved in one patient on postoperative ultrasonography. No reoperation for recurrent ureteral obstruction was documented during follow-up.

**Conclusion:**

Delayed diagnosis of ureteral endometriosis may lead to severe unilateral renal impairment and even the need for nephrectomy. Normal serum creatinine levels do not exclude severe dysfunction of the affected kidney. Early urinary tract imaging, split renal function assessment, individualized surgery, and long-term follow-up are essential for renal preservation.

## Highlights

- This 23-year retrospective case series summarizes patients with severe renal impairment caused by ureteral endometriosis and highlights the need for early recognition by gynecologists and urologists.

## Introduction

Endometriosis is a condition in which functional endometrial tissue grows and infiltrates tissues outside the uterine cavity. It can occur in multiple systems both within and beyond the pelvis, affecting 5–10% of women of reproductive age worldwide (1). The prevalence of urinary tract endometriosis is approximately 1–12%, although it can reach 16.4–52.6% in patients with deep infiltrating endometriosis (2, 3). There have been many reports on patients with ureteral endometriosis; however, these patients are prone to misdiagnosis and delayed diagnosis and treatment due to subtle and non-specific early symptoms, resulting in prolonged intervals between symptom onset and diagnosis. Many patients may have renal dysfunction or a non-functional affected kidney requiring surgical removal (4). This study aimed to summarize the clinical characteristics, surgical management, and follow-up outcomes of patients with ureteral endometriosis and severe impairment of the affected kidney, with the goal of improving awareness, promoting early diagnosis, and facilitating renal preservation.

## Materials and methods

### Study design and patients

A retrospective descriptive case series was conducted using data from patients who underwent surgery for endometriosis at Peking University First Hospital between January 2000 and January 2022. Given the long study period, imaging availability, surgical approaches, and multidisciplinary management strategies varied among cases. Therefore, this analysis was designed as a descriptive case series rather than an era-based comparative study.

Preoperative diuretic renal dynamic imaging identified 15 patients with severe renal impairment, and postoperative pathology confirmed ureteral endometriosis in all cases. All included patients had ureteral stenosis and severe hydronephrosis caused by ureteral endometriosis. Clinical history, preoperative examination findings, operative records, pathological findings, and follow-up data were retrospectively extracted from the hospital medical record system.

### Preoperative assessment

Before surgery, patients underwent routine laboratory tests, urinary tract ultrasonography, gynecological ultrasonography, and diuretic renal dynamic imaging to evaluate renal function. Pelvic magnetic resonance imaging and/or urinary tract computed tomography were performed when available or clinically indicated to further assess the extent of pelvic endometriosis, ureteral obstruction, hydronephrosis, and renal atrophy.

Severe renal impairment was defined as an affected-kidney glomerular filtration rate (GFR) of less than 15 mL/min on diuretic renal dynamic imaging. Serum creatinine was also recorded, but it was interpreted as an indicator of overall renal function rather than the split renal function of the affected kidney.

Double-J ureteral stenting or percutaneous nephrostomy was not routinely performed in all patients. The decision to perform preoperative urinary drainage was individualized based on the feasibility of retrograde stenting, severity of obstruction, urinary symptoms, suspected salvageability of the affected renal unit, and multidisciplinary assessment. Percutaneous nephrostomy was performed when retrograde double-J stenting failed or was technically infeasible and/or when urgent decompression was considered necessary.

### Surgical decision-making

Surgical management was individualized after preoperative assessment by the gynecologic and urologic teams. The choice of ureterolysis, ureteroneocystostomy, or nephrectomy was based on an integrated assessment of affected kidney function on diuretic renal dynamic imaging, radiologic evidence of renal atrophy and cortical thinning, the severity and location of ureteral obstruction, feasibility of preoperative urinary drainage, suspected extrinsic or intrinsic ureteral involvement, fertility preservation requirements, and intraoperative findings.

Ureterolysis was selected when the affected renal unit was considered potentially salvageable and the ureteral obstruction appeared to be primarily caused by extrinsic compression or fibrotic adhesions, with the possibility of restoring ureteral patency after careful dissection. Ureteroneocystostomy was performed when the distal ureter showed fixed, severe stenosis, when intrinsic ureteral involvement was suspected, or when ureterolysis alone was insufficient to restore a patent and viable ureter. Nephrectomy was considered when the affected kidney was judged to be non-salvageable because of extremely poor split renal function, substantial renal atrophy, long-standing severe obstruction, and a low expected likelihood of functional recovery after decompression or reconstruction.

### Operative strategy and surgical procedures

Surgery was performed by experienced gynecologic surgeons, with urologic surgeons involved when ureteral reconstruction, nephrectomy, or complex urinary tract management was required. The surgical route was selected according to disease extent, prior surgical history, severity of pelvic adhesions, and the need for combined urologic procedures.

After abdominal entry, pelvic adhesions, ovarian endometriomas, uterosacral ligament involvement, rectovaginal lesions, bladder involvement, parametrial or paracervical fibrotic lesions, and ureteral obstruction were systematically evaluated. The revised American Fertility Society classification was used intraoperatively to stage endometriosis when gynecologic surgery was performed.

For ureteral endometriosis, the ureter was first identified proximal to the suspected obstructive lesion and then carefully dissected distally along its course. Ureterolysis was performed by separating the ureter from the surrounding fibrotic or endometriotic tissue until the compressed or stenotic segment was released and ureteral patency was considered restored. During dissection, particular attention was paid to the relationship between the ureter, uterosacral ligament, pelvic sidewall, and parametrial or paracervical lesions.

When fixed distal ureteral stenosis, suspected intrinsic ureteral involvement, or inadequate restoration of ureteral patency after ureterolysis was identified, ureteral resection and ureteroneocystostomy were performed. Nephrectomy was performed in patients whose affected kidney was considered non-salvageable based on multidisciplinary assessment.

Concomitant gynecologic procedures were performed according to lesion distribution, disease severity, and fertility preservation requirements. These procedures included ovarian endometrioma cystectomy, oophorectomy, salpingectomy, total hysterectomy, excision of uterosacral ligament lesions, excision of rectovaginal or vaginal nodules, bladder lesion resection, and excision of parametrial or paracervical fibrotic lesions when present.

The operation duration was calculated from the induction of anesthesia to patient emergence from anesthesia. Intraoperative blood loss was estimated based on the volume of blood in the negative-pressure suction container and the number of gauzes used.

### Follow-up

Postoperative follow-up included urinary tract ultrasonography, urinalysis, and gynecological ultrasonography at 1, 6, and 12 months after surgery when these assessments were available. Outpatient visits and/or telephone interviews were used to assess urinary symptoms, endometriosis-related pain, pregnancy status, and recurrence. Patients were considered lost to follow-up if no outpatient records was available and they could not be reached by telephone after repeated attempts.

### Statistical analysis

Considering the small sample size and the descriptive nature of this case series, no inferential statistical comparisons were planned. Normally distributed continuous variables are presented as means ± standard deviations, whereas skewed continuous variables are presented as medians and ranges. Categorical variables are reported as absolute numbers and percentages.

## Results

### Patient characteristics

A total of 15 patients with ureteral endometriosis and severe impairment of the affected kidney were included in this study. All patients had hydronephrosis and hydroureter confirmed by preoperative imaging. The mean age was 40.1 ± 6.0 years, and the mean body mass index was 24.3 ± 4.5 kg/m^2^. Eleven patients (73.3%) had a history of childbirth, three (20.0%) had primary infertility, and six (40.0%) had a history of previous surgery for ovarian endometriosis.

Three patients (20.0%) were asymptomatic at presentation, whereas the remaining 12 patients (80.0%) had endometriosis-related pain and/or urinary symptoms. The most common symptoms were dysmenorrhea and lumbar discomfort. Detailed baseline characteristics and presenting symptoms are summarized in Table 1.

**Table 1:** Patient characteristics (*n* = 15).

Patient characteristic	Value, *n* (%) or mean ± SD
Age (years)	40.1 (±60)
Body mass index (kg/m^2^)	24.3 (±4.5)
Parity	
Nulliparous	4 (26.7%)
Para>1	11 (73.3%)
Infertility	
No infertility	11 (73.3%)
Primary infertility	3 (20.0%)
Secondary infertility	0 (0)
Unknown infertility	1 (6.7%)
Cesarean	4 (26.7%)
Previous surgery for endometriosis	
Transabdominal	6 (40%)
Translaparoscopic	0 (0)
Presenting symptoms	
None	3 (20%)
Dysmenorrhea	9 (60%)
Non-menstrual pain	3 (20%)
Dyspareunia	1 (6.7%)
Anal bulge	1 (6.7%)
Lumbar discomfort	7 (46.7%)
Urinary symptoms	
Pollakisuria	1 (6.7%)
Urgency	0 (0)
Dysuria	1 (6.7%)
Hematuria	4 (26.7%)
Hydronephrosis	15 (100%)
Renal atrophy	8 (53.3%)

### Preoperative assessment

Diuretic renal dynamic imaging showed an affected-kidney GFR of <15 mL/min in all 15 patients, including four patients (26.7%) with an affected-kidney GFR of 0 mL/min. In contrast, only one patient had elevated serum creatinine levels, whereas the remaining 14 patients had normal serum creatinine levels. This discrepancy was considered to be related to the preserved compensatory function of the contralateral kidney, as serum creatinine indicates overall renal function rather than the split renal function of the affected kidney.

CA125 was measured in 11 patients, and five had elevated CA125 levels. Preoperative double-J ureteral stents were placed in five patients, and three patients underwent percutaneous nephrostomy after retrograde stent placement had failed or was technically infeasible. Other preoperative laboratory and imaging findings are shown in Table 2.

**Table 2:** Auxiliary examination.

Examination item	Value, *n* (%) or mean ± SD
Blood creatinine (*n* = 15)	
Abnormality	1 (6.7%)
Normality	14 (93.3%)
Average value	89.7 (±17.5)
Cancer Antigen 125 (CA 125) (*n* = 11)	
Abnormality	5 (45.5%)
Normality	6 (54.5%)
Average value	68.1 (±69.6)
Urine red blood cell count (*n* = 15)	
0–3/HP	9 (60%)
>3/HP	6 (40%)
Pelvic ultrasonography	12 (80%)
Renal ultrasonography	15 (100%)
Magnetic resonance imaging	7 (46.7%)
Urinary tract computed tomography	9 (60%)
Diuretic renal dynamic imaging	15 (100%)

### Surgical findings and procedures

Nine patients (60.0%) underwent laparoscopic surgery, and six patients (40.0%) underwent laparotomy. Among the 12 patients who underwent gynecologic surgery and were eligible for staging according to the revised American Fertility Society classification, eight (66.7%) had stage IV disease, two (16.7%) had stage III disease, and two (16.7%) had stage II disease. The mean operative time was 239.2 ± 98.3 min, and the mean estimated blood loss was 252.7 ± 297.8 mL.

Regarding ureteral procedures, three patients (20.0%) underwent ureterolysis, four (26.7%) underwent ureteroneocystostomy, and eight (53.3%) underwent nephrectomy because the affected kidney was considered non-salvageable. Concomitant gynecologic procedures were performed according to the disease distribution and fertility preservation requirements, including ovarian endometrioma cystectomy, oophorectomy, total hysterectomy, excision of uterosacral ligament lesions, excision of rectovaginal or vaginal nodules, and bladder resection when indicated. Detailed intraoperative findings and associated procedures are presented in Table 3.

**Table 3:** Intraoperative findings.

Intraoperative finding or procedure	Value, *n* (%) or mean ± SD
Duration of surgery (min)	239.2 (±98.26)
Blood loss (mL)	252.7 (±297.8)
rAFS classification (*n* = 12)	
Stage I	0
Stage II	2 (16.7%)
Stage III	2 (16.7%)
Stage IV	8 (66.7%)
Sites of concomitant endometriosis	
Endometrioma	
Left	7 (46.7%)
Right	7 (46.7%)
Bilateral	1 (6.7%)
Uterosacral ligaments	
Left	3 (20%)
Right	3 (20%)
Bilateral	1 (6.7%)
Vaginal posterior fornix	6 (40%)
Bladder	1 (6.7%)
Bowel	0
Ureteral procedures	
Ureterolysis	3 (20%)
Ureteral anastomosis	0
Ureteroneocystostomy	4 (26.7%)
Nephrectomy	8 (53.3%)
Associated procedures	
Ovarian endometrioma cystectomy	3 (20%)
Oophorectomy	8 (53.3%)
Total hysterectomy	10 (66.7%)
Excision of uterosacral ligaments	6 (40%)
Excision of rectovaginal nodule	6 (40%)
Excision of vaginal nodule	1 (6.7%)
Bladder resection	1 (6.7%)
Surgical route	
Laparoscopy	9 (60%)
Transabdominal	6 (40%)
Intraoperative complications	2 (13.3%)

Two patients experienced intraoperative complications related to severe adhesions. One patient had major bleeding, and another sustained a bowel injury requiring partial small-bowel resection.

### Follow-up outcomes

Thirteen patients (86.7%) were successfully followed up through outpatient visits and/or telephone interviews, with a median follow-up duration of 61 months (range, 25–180 months). The remaining two patients were lost to follow-up because they could not be contacted despite repeated telephone attempts after discharge; therefore, the long-term postoperative outcome analysis was based on the 13 patients with available follow-up data.

Postoperatively, double-J ureteral stents were successfully removed at 3 months after surgery in patients who underwent kidney-preserving procedures, whereas patients who underwent nephrectomy did not require further ipsilateral stent management. In the three patients with preoperative nephrostomy drainage, the nephrostomy tubes were clamped at 2 weeks after surgery and removed at 2 months postoperatively.

Among the seven patients who underwent kidney-preserving procedures, postoperative urinary tract ultrasonography showed disappearance of hydronephrosis in six patients and significant improvement in one patient. Postoperative diuretic renal dynamic imaging was not routinely performed; therefore, postoperative split renal GFR of the preserved kidney was not systematically available.

Nine patients (60.0%) received postoperative gonadotropin-releasing hormone agonist (GnRH) therapy for 3–6 cycles. During follow-up, endometriosis-related pain and urinary symptoms improved in the majority of patients. Two patients still reported mild lumbar discomfort, and one reported left lower abdominal pain, although these symptoms had improved compared with their preoperative status. One patient conceived successfully 6 months after surgery.

## Discussion

Ureteral endometriosis is relatively uncommon, but its reported incidence has increased in recent years, probably because of improved imaging techniques and greater clinical awareness (5, 6). Ureteral involvement may result from direct infiltration of endometriotic tissue into the ureteral wall or from extrinsic compression and fibrosis around the ureter, leading to ureteral deviation, stenosis, hydroureter, hydronephrosis, and eventually loss of renal function. Importantly, ureteral endometriosis may be clinically silent or present with non-specific symptoms, and the absence of urinary symptoms or abnormal serum creatinine does not rule out ureteral involvement. Although hydronephrosis may be absent in some early or less advanced cases, ureteral distortion, medial deviation, or stenosis can already be present and may be detected by careful imaging assessment (7, 8). Delayed diagnosis may lead to irreversible renal impairment, and 25–43% of patients have been reported to have hydronephrosis or renal dysfunction at the time of diagnosis (9).

Previous studies have reported that ureteral endometriosis is usually unilateral and more frequently affects the distal ureter, often in association with ovarian endometrioma, uterosacral ligament disease, parametrial involvement, or rectovaginal deep endometriosis (10–14). In this cohort, patients were older than the peak age reported in the literature, with a mean age of 40.1 years. This could be due to delayed detection of ureteral involvement before the development of severe kidney impairment. In addition, laterality did not show a clear left-sided predominance in our cohort, probably because of the small sample size and the inclusion of only patients with severe renal impairment.

An important finding of our study was that serum creatinine was insensitive for detecting severe unilateral renal impairment. Although all patients had an affected-kidney GFR of <15 mL/min on diuretic renography, only one patient had elevated serum creatinine. This can be explained by compensation from the contralateral kidney in patients with unilateral obstruction. Therefore, normal serum creatinine does not rule out severe loss of function in the obstructed kidney. In patients with suspected ureteral endometriosis, especially those with hydronephrosis or hydroureter on imaging, split renal function should be assessed by diuretic renography rather than by relying only on serum creatinine.

Recent literature has shifted the focus of early diagnosis from detecting hydronephrosis alone to systematically assessing the course of the ureter. Previous studies have emphasized urinary tract ultrasonography in patients with deep endometriosis, particularly when rectovaginal nodules, uterosacral ligament lesions, ovarian endometrioma, or periureteral lesions are present (6, 15). More recently, Carfagna et al. have reported that transvaginal ultrasound assessment of ureteral medial deviation secondary to deep endometriosis may help predict the need for ureterolysis during laparoscopic surgery (8). This concept is clinically important because ureteral medialization caused by parametrial or periureteral fibrosis may be present even before significant ureteral dilatation or hydronephrosis develops. Therefore, in patients with posterior compartment deep endometriosis, uterosacral ligament lesions, parametrial fibrosis, rectovaginal nodules, or ovarian endometrioma, transvaginal ultrasound should not only evaluate pelvic lesions but also trace the distal ureteral course and assess possible ureteral medialization (8).

When urinary tract ultrasonography shows hydroureter or hydronephrosis, further assessment of split renal function is necessary (11, 13). Diuretic renography is particularly useful because it provides a functional assessment of the affected renal unit and can identify severe unilateral renal impairment that may be missed by serum creatinine. Pelvic magnetic resonance imaging (MRI) and computed tomography (CT) urography may further help define the extent of deep endometriosis, the site of obstruction, renal atrophy, and the anatomical relationship between endometriotic lesions and the ureter (16, 17).

Medical therapy may relieve endometriosis-related pain and suppress disease activity (18), but it cannot resolve established periureteral fibrosis or fixed ureteral stenosis (5). Therefore, surgery remains the main treatment for ureteral endometriosis with obstruction or renal impairment (19–21). The goals of surgery are to relieve urinary obstruction, preserve renal function when possible, remove associated endometriotic lesions, improve symptoms, reduce recurrence, and minimize complications. The choice of surgical procedure should be individualized according to the type and severity of ureteral involvement (20, 21).

In this cohort, surgical decision-making was individualized because all patients had already developed severe kidney impairment by the time of diagnosis. The choice between ureterolysis, ureteral reimplantation, and nephrectomy therefore depended not only on the numerical GFR value but also on the salvageability of the affected renal unit, the degree of renal atrophy, the location and severity of ureteral stenosis, and the intraoperative feasibility of restoring a viable ureter. This is particularly important because a severely impaired unilateral kidney may coexist with normal serum creatinine when the contralateral kidney maintains adequate compensatory function.

The anatomical classification proposed by Ianieri et al. provides a useful framework for tailoring surgical strategy according to the depth and complexity of ureteral dissection (22). In this classification, type 1 ureterolysis involves dissection of periureteral endometriotic or fibrotic tissue without opening the presacral fascia; type 2 requires incision or partial excision of the presacral fascia to mobilize the ureter; and type 3 involves dissection at the level of the ureteral adventitia to remove disease adherent to the ureteral wall. This classification emphasizes that ureterolysis is not a uniform procedure: deeper dissection may increase the risk of ureteral devascularization, residual stenosis, fistula, or the need for ureteral reconstruction (22).

Based on this concept, ureterolysis may be appropriate when obstruction is primarily caused by extrinsic compression or adhesions and the ureter can be safely mobilized with restoration of patency (20, 22, 23). Ureteroneocystostomy should be considered when there is fixed distal ureteral stenosis, suspected intrinsic involvement, poor ureteral viability, or inadequate restoration of ureteral caliber after ureterolysis (24). Nephrectomy may be necessary when the affected kidney is considered non-salvageable because of extremely poor split renal function, significant renal atrophy, long-standing obstruction, and a low likelihood of functional recovery (25).

Currently, laparoscopic surgery is the preferred approach for many patients with deep endometriosis because it provides magnified visualization of pelvic sidewall anatomy and facilitates precise dissection around the ureter (26, 27). However, in patients with severe adhesions, extensive disease, or a non-salvageable kidney requiring nephrectomy, the surgical route and extent of surgery should be individualized. Management should also address associated pelvic endometriotic lesions, including ovarian endometrioma, uterosacral ligament disease, rectovaginal nodules, parametrial or paracervical lesions, and bladder involvement when present (10, 14, 27). Postoperative hormonal treatment and long-term follow-up should be tailored according to symptoms, fertility requirements, residual disease, and the risk of recurrence. Follow-up should include assessment of symptoms, gynecologic examination, urinary tract imaging, and renal function evaluation (19).

This study has several limitations. First, it was a retrospective single-center case series with a small sample size and no control group. Second, the study period was long, spanning from 2000 to 2022. During this period, diagnostic imaging, surgical expertise, and multidisciplinary management of ureteral endometriosis evolved substantially. Earlier in the study period, ureteral involvement may have been less systematically screened, whereas more recent practice has increasingly emphasized urinary tract ultrasonography, pelvic MRI, CT urography, and renal functional assessment in patients with deep endometriosis. These changes may have influenced the timing of diagnosis and may partly explain why some patients in this cohort presented with advanced renal damage. In parallel, improvements in laparoscopic deep endometriosis surgery and closer gynecology-urology collaboration may have affected surgical decision-making, including the choice between ureterolysis, ureteral reconstruction, and nephrectomy. Due to the small sample size, we could not perform a reliable era-based comparison. Therefore, temporal heterogeneity should be considered when interpreting patient selection, treatment distribution, and outcomes. Third, postoperative diuretic renography was not routinely repeated in all kidney-preserving patients. Therefore, although postoperative ultrasonography showed resolution or improvement of hydronephrosis, we could not quantitatively evaluate the recovery of split renal function in the preserved kidneys. Finally, because operative and pathological reports did not consistently document the depth of ureterolysis or the precise depth of ureteral wall involvement, we could not reliably classify all cases using the Ianieri ureterolysis classification or perform a subgroup analysis based on intrinsic versus extrinsic ureteral involvement. Future studies should include standardized preoperative ureteral imaging, detailed surgical classification, and postoperative renal functional assessment to better evaluate diagnosis, surgical strategy, and renal recovery in ureteral endometriosis.

## Conclusion

Ureteral endometriosis may remain clinically silent until severe unilateral renal impairment has developed. Normal serum creatinine levels do not exclude advanced dysfunction of the affected kidney when contralateral renal function is preserved. Therefore, patients with suspected deep endometriosis, especially those with posterior compartment lesions or urinary tract dilatation, should undergo timely urinary tract imaging and assessment of split renal function. Individualized surgical management involving gynecologic and urologic teams is essential to relieve obstruction, preserve renal function when possible, and avoid delayed nephrectomy. Long-term urinary tract follow-up is necessary after kidney-preserving surgery.

## Data Availability

The original contributions presented in the study are included in the article/supplementary material, further inquiries can be directed to the corresponding author.
